# A Capillary Computing Architecture for Dynamic Internet of Things: Orchestration of Microservices from Edge Devices to Fog and Cloud Providers

**DOI:** 10.3390/s18092938

**Published:** 2018-09-04

**Authors:** Salman Taherizadeh, Vlado Stankovski, Marko Grobelnik

**Affiliations:** 1 Artificial Intelligence Laboratory, Jozef Stefan Institute, Ljubljana 1000, Slovenia; 2 CVS Mobile d.d., Ljubljana 1000, Slovenia; 3 Faculty of Civil and Geodetic Engineering, University of Ljubljana, Ljubljana 1000, Slovenia; 4 School of Computing and Information Systems, University of Melbourne, Melbourne, VIC 3010, Australia

**Keywords:** Internet of Things, container-based virtualization, Microservices, Edge computing, Fog computing, on/offloading

## Abstract

The adoption of advanced Internet of Things (IoT) technologies has impressively improved in recent years by placing such services at the extreme Edge of the network. There are, however, specific Quality of Service (QoS) trade-offs that must be considered, particularly in situations when workloads vary over time or when IoT devices are dynamically changing their geographic position. This article proposes an innovative capillary computing architecture, which benefits from mainstream Fog and Cloud computing approaches and relies on a set of new services, including an Edge/Fog/Cloud Monitoring System and a Capillary Container Orchestrator. All necessary Microservices are implemented as Docker containers, and their orchestration is performed from the Edge computing nodes up to Fog and Cloud servers in the geographic vicinity of moving IoT devices. A car equipped with a Motorhome Artificial Intelligence Communication Hardware (MACH) system as an Edge node connected to several Fog and Cloud computing servers was used for testing. Compared to using a fixed centralized Cloud provider, the service response time provided by our proposed capillary computing architecture was almost four times faster according to the 99th percentile value along with a significantly smaller standard deviation, which represents a high QoS.

## 1. Introduction

With the emergence of the Internet of Things (IoT), various Artificial Intelligence (AI) algorithms of different computational complexities are being designed to operate on continuously generated sensor data streams. Many new smart IoT applications, for example, for self-driving cars, are time-critical in nature and need to address specific Quality of Service (QoS) requirements including low communication latency, fast computations and high bandwidth. In order to address such requirements, the centralized Cloud computing paradigm has evolved towards new promising distributed Edge and Fog computing trends [1,2].

While the terms Edge and Fog computing have been used by various researchers with slightly different meanings [3], in this study we refer to an Edge node as a computing device which has a processor or multi-processor on-board system. Examples are battery-driven vehicles, robots, smartphones, Raspberry Pi [4] or Arduino [5]. In this context, Fog nodes can be understood as Cloud computing infrastructures, which exist in the close geographic proximity of Edge nodes. To this end, different lightweight operating systems such as RancherOS [6] or CoreOS [7] make it possible to turn even small devices such as routers into Cloud providers, in which case they are considered to be Fog computing infrastructure.

The Edge/Fog computing model is a new computing paradigm aiming to optimize smart applications to expand their functionalities close to the geographic location of the IoT devices, rather than outsourcing computations to far away datacenters.

In an advanced Edge/Fog computing framework, modern software engineering approaches can be made to exploit lightweight Microservices [8] packaged into containers to achieve a high degree of automation, deployment, elasticity and reconfiguration of smart IoT applications at runtime. To this end, various container management and orchestration technologies have emerged, including Docker [9], Kubernetes [10], OpenShift Origin [11] and Swarm [12]. In other words, once these technologies are combined with the Microservices architecture, a great level of agility in development, deployment and reconfiguration of applications can be achieved.

Although many isolated computing technologies already exist, currently there is a significant lack of computing architectures and solutions which combine the aforementioned new technologies together to monitor and address QoS requirements for emerging smart IoT applications. This study particularly aims to design a new distributed computing architecture that can support smart applications under varied number of workloads in which IoT devices dynamically move from one geographic location to another. The goal is to provide enough computational capabilities required by AI and other computationally intensive tasks to process the data generated by such IoT devices. Another requirement is to use existing open standards when designing and implementing this new architecture.

An additional goal of this study is to demonstrate the feasibility of the new architecture on a smart telematics application [13]. The application involves the use of a Motorhome Artificial Intelligence Communication Hardware (MACH) [14] system, which is deployed in a vehicle. All software services required to support the system are implemented as container-based Microservices. This includes functionalities of the MACH system that are able to observe driving dynamics, such as acceleration, braking, turning and so on, perform real-time analytics and trigger alerts to the fleet manager for situations such as aggressive steering maneuvers, where possible accidents may occur.

Over the entire course of this study, the following research questions were addressed:

- What potential monitoring metrics can impact the performance of emerging smart IoT applications, and how can these quality parameters be monitored, exchanged and used?
- What architectural approach is needed to be able to offload requests from Edge to Fog or Cloud under a varied number of workloads in which IoT devices dynamically move from one geographic location to another?
- From the interoperability viewpoint, what are the main existing open standards which can be used to design a capillary distributed computing architecture that is able to support IoT applications?

The rest of the paper is organized as follows. Section 2 presents a summary of background and related works, including a State-of-the-Art review of modern Edge and Fog computing frameworks. Section 3 introduces our proposed capillary computing architecture and its implementation. Key QoS parameters used by the proposed architecture for runtime autonomic orchestration of container-based Microservices is illustrated in Section 4. Section 5 presents empirical evaluation along with experimental results. Finally, the conclusion appears in Section 6.

## 2. Background and Related Works

Over the past few years, the DevOps concept has emerged with the promise of radical improvements in the application life-cycle through the development of dependable, reusable software components called Microservices. The concept of Microservices provides a revolutionary architecture, which relies mainly on new lightweight container technologies for virtualization. This design makes it possible to develop new distributed computing software systems that are able to achieve high QoS, flexibility, dependability and other properties due to their autonomic self-behavior, such as self-monitoring, self-adaptation, self-reconfiguration, self-optimization, self-healing, self-regulating and so on. Recently developed State-of-the-Art Edge and Fog architectures aim at providing such advanced properties to smart IoT applications. Our new capillary distributed computing architecture is compared to these developments, as explained in the next subsections.

### 2.1. The Microservices Architecture

The current DevOps movement intends to make remarkable progress in the software lifecycle. Instead of delivering monolithic, stand-alone applications, modern software engineering workbenches such as mOSAIC [15], Juju [16] or SWITCH [17] stand for small, discrete, reusable, elastic, well-tested software components. Such interdependent software components are individually built as Microservices, where each service is responsible for its own small objective.

**Figure 1:**
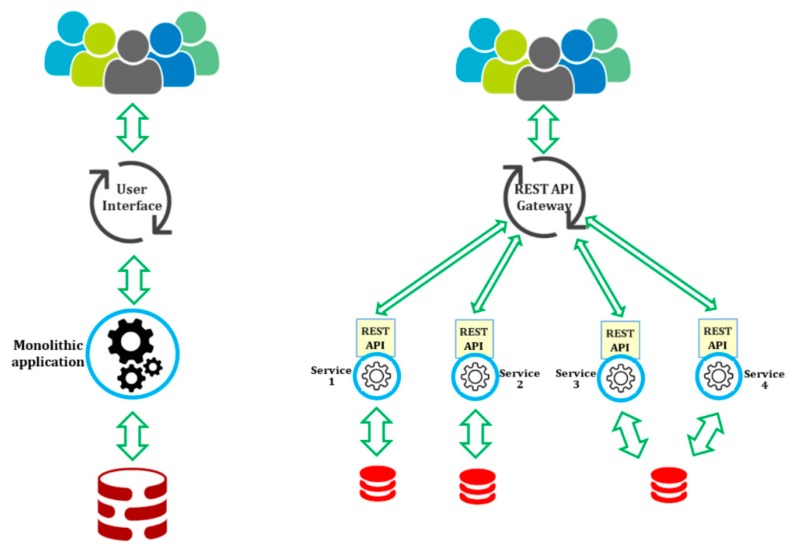
Applications structure of monolithic versus Microservices architecture.

Figure 1 compares the Microservices architecture to the usual monolithic application design. In the monolithic design, all functional logic for handling requests runs within a single process. An important disadvantage is that even a very tiny update made to a small segment of the application requires the entire monolith to be re-developed and then re-deployed again. In the Microservices architecture, each business capability is a self-contained service with a well-defined REST Application Programming Interface (API). It should be noted that each Microservice running on a specific host can be easily managed at runtime that includes various operations such as deployment, instantiation, movement, replication, termination, and so on.

### 2.2. Container-Based Microservices

Hypervisor-based virtualization technologies [18] are able to support standalone Virtual Machines (VMs) which are independent and isolated from the host machine. Each VM instance has its own operating system and a set of libraries, and operates within an emulated environment provided by the hypervisor. This makes VMs heavy to manage. In this context, container-based technologies are considered to be more lightweight [19]. Compared to VMs, the use of containers does not require an operating system to boot up that gains an increasing popularity in the Edge, Fog and Cloud computing domains [20]. Resource usage of VMs is extensive [21], and thus typically, they cannot be easily developed on small servers or resource-constrained devices, such as Raspberry Pis or routers. In contrast, containers can be used flexibly in such frameworks. Since their nature is lightweight, deployment of container-based services at runtime can be achieved faster than VMs [22]. Due to these advantages, various container-based virtualization platforms, such as the Google Container Engine (GCE) [23] and the Amazon EC2 Container Service (ECS) [24] have emerged as suitable alternatives to hypervisor-based virtualization. Table 1 provides a comparison between container-based and VM-based virtualization [25].

Container-based virtualization is supported by various operating systems such as RancherOS and CoreOS, orchestration technologies such as Kubernetes and Swarm, as well as schedulers such as Mesos [26]. These technologies are now provided readily in datacenters, micro-datacenters and standalone devices such as routers and Raspberry PIs. They are particularly suitable for designing smart IoT applications based upon Edge and Fog computing architectures.

In summary, container-based virtualization technologies together with the Microservices architecture provide a modern, agile computing framework for time-critical applications in highly dynamic runtime environments where Microservices can be easily started and stopped as driven by various events.

### 2.3. Edge/Fog State-of-The-Art Review

Existing Edge and Fog computing architectures are designed with the aim of achieving high QoS operation. Smart IoT applications usually require the use of various streaming sensor data, and need significant processing capacity for diverse AI algorithms. To this end, different aspects need to be taken into account when designing suitable Edge and Fog computing architectures These include the dynamic nature of IoT devices (e.g., whether they are moving or static), type of Big Data problems (e.g., velocity, veracity, variety, volume), computational complexity of data analytics, and similar. Furthermore, the workload can significantly change depending on various events in the runtime environment [27]. This requires new elasticity mechanisms to be used at the Edge of the network. Along these lines, offering desirable application performance provided by Edge and Fog computing frameworks has been the goal of several studies.

The existing approaches to the definition of a suitable Edge or Fog computing architecture that we found in the literature are hereby categorized into three groups: (i) studies that use a static amount of Edge and Fog resources as a complete replacement to Cloud infrastructures; (ii) studies that intend to discover available Edge and Fog computing resources during runtime, when the workload increases and elasticity must be achieved; and (iii) replicating services in all Edge devices, Fog nodes and Cloud resources to address reliability and resilience problems. These are discussed in the following.

- Using a static amount of Edge and Fog resources as a complete replacement to Cloud infrastructures: The best practice in such a solution is exploiting general-purpose Edge nodes that can be involved in heterogeneous types of computation and data analytics. To this end, the LightKone project [28] is recently aimed at moving computation and data storage completely out of the Cloud and directly on the extreme Edge of the network. As another example, the main objective of Open Edge Computing (OEC) project [29] is enabling all nearby Edge components such as Wi-Fi access points, DSL-boxes, base stations to offer computing and storage resources through standardized, open mechanisms to any types of applications. The OEC solution enables an improvement in customer experience through low latency interaction with compute and storage resources just one hop away from the end-users. Similarly, ParaDrop [30] is also proposed that only considers using Edge and Fog nodes as the replacement to the centralized Cloud. Such projects focus mainly on offering distributed orchestration frameworks through which Edge and Fog resources can be assigned to running services. Such solutions can be also extended to exploit Edge-based distributed caching policies [31], which may increase the chance to react to runtime fluctuations in the workload before a performance issue arises. However, achieving the goal to have all Edge and Fog nodes as general-purpose computation and storage resources is still difficult. This is because, at first, such static Edge resources cannot be virtualized as they have physical items such as attached antennas, and hence they may not be scalable enough to handle increasing workloads at runtime.
- Discovering other available Edge and Fog computing resources during runtime, when the workload increases and elasticity must be achieved: Di Salle et al. [32] proposed a new software architecture to develop IoT systems of new generation. This architecture is highly capable of connecting smart devices with each other in order to combine services provided not only from the Cloud and network resources, but also by Things themselves. Exploiting the Edge of the network requires discovery methods to find available Edge and Fog nodes which can be leveraged in a distributed computing environment [33,34,35]. Resource discovery methods employed in the Cloud environment cannot be useful in this context for the discovery of Edge and Fog nodes. This is because Edge nodes usually are in a private network, and hence organizations should discuss regulations for using their own resources with those who may exploit these devices [36]. In this regard, Zenith [37] as a resource allocation model allows Edge and Fog infrastructure providers to establish resource sharing contracts with service providers at runtime. Consideration of multi-tenancy issues on Edge and Fog nodes comes first in such environments in which the Edge and Fog resource provider and each of customers should have different views on these infrastructures [38]. Besides that, it is not an easy task to convince other entities to make use of their own resource in order to enhance computing or storage capabilities at runtime [39]. It should be also noted that volunteer resources are generally less reliable and less predictable since they may leave the execution environment at any time.
- Replicating services in all Edge devices, Fog nodes and Cloud resources to address reliability and resilience problems: This approach includes replicated services running not only on Edge and Fog resources close to the users, but also on Cloud infrastructure [40,41,42,43] which means resources are wasted when the workload drops. In this context, if Edge and Fog resources allocated to process incoming requests are overloaded, and hence these nodes are no longer capable of improving the application QoS, additional arrived requests will be sent to the application server running on the Cloud. However, replication of servers comes with its own technical challenges. For example, temporary inconsistencies among storage or computing replicas are required to be taken into account. Moreover, different organizations have various regulations of using computing and storage infrastructures such as legislation on the geographic location of service instances or data storage servers [44].

**Figure 2:**
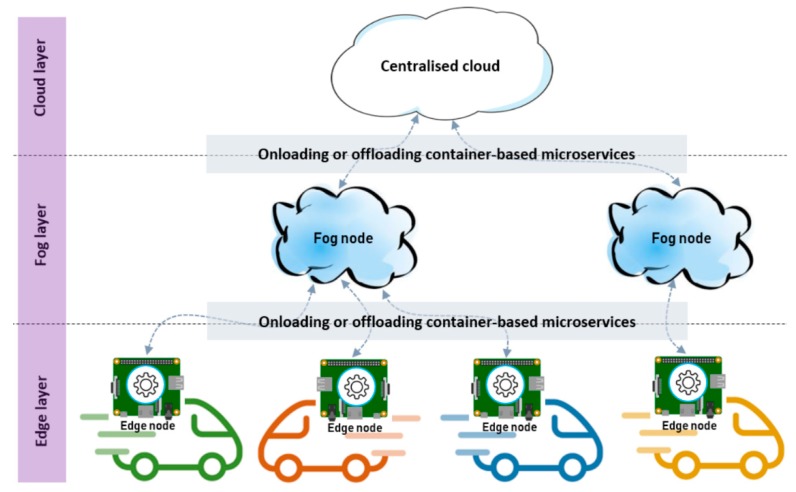
Onloading or offloading Microservices between different layers (Edge, Fog and Cloud) in the proposed capillary distributed computing architecture.

Our analysis of differences and similarities among existing Edge and Fog computing architectures has provided us with necessary insights to propose an innovative, capillary distributed computing architecture which addresses computational problems of smart IoT applications, as shown in Figure 2.

These scenarios involve moving IoT devices, such as cars and robots, in which case computation and storage service may need to be moved from one Edge node to another one. In other words, our new architecture is capable of managing highly dynamic environments at runtime in situations when IoT devices are moving from one geographic location to another one because it is necessary to move container-based Microservices from one Edge node to another. Moreover, the newly proposed capillary architecture is able to offload requests from an Edge node to a Fog or Cloud resource when the Edge node is overloaded and it has a limited amount of computation power or storage capacity at runtime. The offloading of containers resembles a capillary process, and hence this is why the proposed architecture is called capillary. For this reason, our proposed solution dynamically provisions Fog or Cloud resources, on which container-based Microservices can be deployed at runtime, whenever elasticity is required. The design takes into consideration all infrastructure, application and network-based parameters, and it is also capable of releasing unnecessary Cloud-based resources in order to offer services at the Edge and Fog computing layer to provide fast response time and avoid wasting costly Cloud infrastructure.

## 3. Architecture and Design

**Figure 3:**
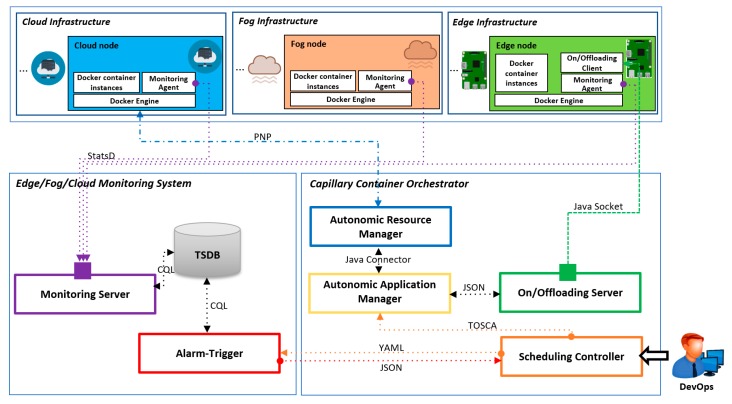
The proposed capillary distributed computing architecture for smart IoT applications.

The Microservices architecture and the existence of various container-based orchestration technologies motivated us to design and develop a new, innovative capillary distributed computing architecture (see Figure 3). Its main purpose is to support computationally and data-intensive applications running at the Edge of the network on resources such as cars, robots, Raspberry PIs, smartphones and similar. The presented capillary distributed computing architecture follows a widely accepted reference model called MAPE-K (Monitor-Analyze-Plan-Execute over a shared Knowledge) applied in different autonomic self-adaptive computing systems [45,46]. In essence, our proposed architecture which consists of several steps is a classical MAPE-K loop feedback instance aimed to serve as guideline to develop self-adaptive software systems. Monitor describing the running environment provides the input data for Analyzer which supports decision-making on whether self-adaptation is necessary in given circumstances. Planner generates suitable actions to adapt the target system according to supported adaptation mechanisms. Executor where receives the change plan includes the adaptation operation. Knowledge Base is also used to store all information about the environment.

Along these lines, the starting points in the proposed architecture are Edge computing resources on which all necessary lightweight Microservices are deployed. In the case of a moving IoT device from one geographic location to another one, the running container on the current Edge node will be terminated and another instance will be launched on another Edge node in close proximity of the IoT device.

Moreover, in case when there is a limited amount of available resources, such as storage capacity or computing power on the Edge node at runtime, the running Microservice will be offloaded from the Edge node to a specific Fog computing node. Therefore, this architecture is capable of providing the necessary elasticity management of the application in situation where the Edge node is going to be overloaded. This process resembles capillary fluid movement, hence, the name of this new architecture. In comparison with centralized Cloud, the Fog computing model is able to decrease the amount of traffic in the network, and it offers low-latency response time for the service. However, this layer will bring difficulties for resource management and task scheduling. For example, if a Fog node is overloaded due to a drastic increase in the workload, mobility of Microservices from the Fog node to the Cloud also needs to be considered during the execution time.

The proposed architecture includes different components defined in a Capillary Container Orchestrator, an Edge/Fog/Cloud Monitoring System, Edge Infrastructure, Fog Infrastructure and Cloud Infrastructure, described in detail in the following paragraphs:

The Monitoring Agent is able to continuously measure a set of metrics related to infrastructure, network and application performance. Infrastructure-specific metrics are CPU, memory, disk, etc. Network-specific metrics are delay, packet loss, jitter, etc. Furthermore, application-related metrics represent the information about the status of the application such as service response time. The Monitoring Agent periodically sends the monitoring data to the Monitoring Server. It should be noted that when the Monitoring Agent is launched, it will automatically send the Monitoring Server a message to register itself as a new metric stream, and then start collecting metrics and continuously forward the measured values to the Monitoring Server.

In this work, Monitoring Agents have been implemented through our own implemented open-source monitoring system which is freely available at GitHub [47] under an Apache 2 license. In this monitoring system, Monitoring Agents are developed via the non-intrusive StatsD protocol [48] which can be implemented for many different programming languages such as Python, C/C++ and Java.

It should be noted that monitoring time intervals for measurements should be optimally determined. Very short monitoring intervals can have a negative influence on the intrusiveness of Monitoring Agents, while large sampling rates may reduce the accuracy of monitoring data. Therefore, the Monitoring System might easily appear as a performance bottleneck due to imprecise monitoring intervals for measurements, whereas other components are appropriately operational within the execution environment.

The Monitoring Server developed in our previous work [22] is a component which receives the monitoring data sent by Monitoring Agents. This component is capable of forwarding such measured values to the TSDB.

The TSDB component is a database used to store QoS monitoring metrics. For this purpose, the free, open-source Apache Cassandra [49] is employed since this database is optimized for storing time-series data. The monitoring system suitable for IoT-based environments needs the capability for storage of large amount of monitoring metrics, and hence the Cassandra TSDB is used to increase the reliability of the whole system.

The Cassandra TSDB component has its own query language called the Cassandra Query Language (CQL). CQL which is an alternative to the traditional Structured Query Language (SQL) can be considered as a simple interface in order to access Cassandra. CQL adds an abstraction layer to hide implementation details and provides native syntaxes for collections and other common encodings. Language drivers are available for Java (JDBC), Python (DBAPI2), Node.JS (Helenus), Go (GOCQL) and C++.

The DevOps provides requirements for the automatic deployment of containerized Microservices such as minimum resource capacity needed to host each service. In addition, the DevOps defines specific zones for the deployment of containerized services over Edge, Fog and Cloud resources. This is because, for example, organizations and companies have strict regulations about the geographic location of data storage or computing services. Moreover, the DevOps determines constraints such as thresholds for CPU, memory and disk utilization as well as the service response time threshold. For example, it is possible to specify a disk-based constraint that containerized services need to be offloaded from the Edge resource to the Fog layer if the free storage capacity on the Edge node is less than a threshold such as 100 MB. Or as another example, a CPU-based constraint can be defined that containerized services need to be offloaded from the Edge node to the Fog layer if the average CPU utilization during the last minute is over a threshold such as 80%.

In many Cloud resource management systems [50,51,52,53,54,55], thresholds for infrastructure utilization (e.g., CPU and memory usage) are set to the value of 80%. If the value of these thresholds is set closer to 100%, the capillary computing design then has no chance to react in a timely manner to runtime changes in the operational environment before a performance degradation arises. On the other hand, if the value of these thresholds used by the Alarm-Trigger is set to less than 80%, then this may cause unnecessary on/offloading actions, wasting costly Cloud-based resources. If the execution environment (e.g., the workload trend) is very even and predictable, thresholds for the utilization of resources can be pushed higher than 80%.

Here we discuss how the response time threshold should be set in general. In order to make the system avoid any performance drop, the value of response time threshold should be set more than the usual time to process a single job without any issue when the system is not overloaded. In the case that the response time threshold is set very close to the value of the usual time to process a single job, the capillary computing architecture may lead to unnecessary reconfiguration of the application, whereas the system is currently able to provide users an appropriate performance without any threat. Moreover, if the response time threshold is set to be too much larger than the value of the usual time to process a single job, the capillary computing design will be less sensitive to application performance and more dependent on infrastructure utilization.

The Alarm-Trigger component [56] calls an API exposed by the Scheduling Controller to fetch a YAML [57] which includes all constraints representing all thresholds. The Alarm-Trigger component is a rule-based entity which continuously checks the incoming monitoring data against determined constraints and notifies the Scheduling Controller when any of thresholds predefined by the DevOps is violated that means the system is going to experience an abnormal situation. In this situation, the Alarm-Trigger calls another API provided by the Scheduling Controller and sends a POST notification as a JSON to this API.

When any of predefined constraint is met, or in other words any of thresholds is violated, the Scheduling Controller will be called by the Alarm-Trigger component. According to the deployment requirements defined by the DevOps, the Scheduling Controller is able to generate an optimal reconfiguration into TOSCA for describing the application recontextualization across the Edge, Fog and Cloud platforms. In other words, if any application reconfiguration should be accomplished in terms of offloading containerized services from Edge devices to Fog or Cloud resources or vice versa, these reconfiguration actions will be determined in a TOSCA specification. Here, TOSCA is a standard language used to describe a portable model defining the topology of applications and required resources in a resource provider-independent and infrastructure-agnostic way.

The optimal reconfiguration, which includes the list of containers to be instantiated on either Edge, Fog or Cloud, determines hardware properties of resources to host each containerized service in terms of CPU MHz, number of CPUs, memory size, disk size, and so on. Furthermore, it includes the deployment zone for each service. Therefore, the optimal reconfiguration will be converted into a TOSCA specification prepared by the Scheduling Controller and then sent to the Autonomic Application Manager.

The Autonomic Application Manager receives the TOSCA specification which includes the optimal reconfiguration of the application. The Autonomic Application Manager can find out all hardware characteristics of resources as well as the zones where containerized services should be redeployed at runtime via all details prescribed by TOSCA.

In case of offloading services from Edge to Fog or Cloud infrastructure, if more than one possible node can be exploited to host containerized services, the Autonomic Application Manager will choose the best one. To this end, various network-level quality parameters of the path between Edge and possible nodes on the Fog or Cloud should be measured before the re-deployment of services at runtime. This is because network-level QoS metrics including delay, jitter, packet loss, throughput, bandwidth and number of intermediate routers are considered as significant factors for providing a stable application performance. Moreover, the Autonomic Application Manager should take into consideration different infrastructure-level parameters as well. In other words, a server with a limited amount of available CPU, memory and disk resources may not be an appropriate option to be used to host Microservices.

It should be noted that the Autonomic Application Manager has to be also able to recognize situations when a new VM on the Cloud must be acquired, or an existing VM should be released. Therefore, in summary, the Autonomic Application Manager processes TOSCA, chooses the actual target hosts to deploy the containerized services and sends JSON-based reconfiguration instructions to the On/Offloading Server.

The Autonomic Resource Manager is in charge of acquiring and releasing Cloud resources. In essence, this component receives acquiring or releasing requests from the Autonomic Application Manager. To manage Cloud resources, the Autonomic Resource Manager directly interacts with the private or public Infrastructure as a Service (IaaS) APIs to acquire or release VMs through ProActive Network Protocol (PNP) [58]. PNP is a robust communication protocol which binds to a given TCP port at the start-up time of VMs. All incoming communications use this TCP port. When a VM is acquired on the Cloud, one script will be executed at the beginning in order to install both the Monitoring Agent and the Docker Engine on the VM. Similar to Edge resources, Docker’s Remote API [59] should be also enabled by this script on every VM.

The reconfiguration API is exposed by the On/Offloading Server to receive JSON-based reconfiguration instructions sent by the Autonomic Application Manager. The On/Offloading Server receives new reconfiguration instructions from the Autonomic Application Manager. Afterwards, the On/Offloading Server translates these instructions into platform-dependent deployment requests which are then sent to the container control API exposed by the On/Offloading Client.

The On/Offloading Client, which is installed on every Edge node, responds to the On/Offloading Server’s requests to start or stop container instances. The container control API is exposed by the On/Offloading Client able to receive requests from the On/Offloading Server. Such requests issued by the On/Offloading Server can be terminating or instantiating containerized Microservices. The On/Offloading procedure’s Java source code is kept publicly available as a third-party tool on GitHub [60].

The developed On/Offloading Client works based on Docker’s Remote API to instantiate new containers or terminate running containers. In essence, Docker’s Remote API is exploited for communication with Docker daemon, which is the core module of the Docker virtualization platform, and it controls the status of containers. On every Edge resource, Fog node or Cloud-based infrastructure, the Docker engine should be installed, and Docker’s Remote API should be exposed.

**Figure 4:**
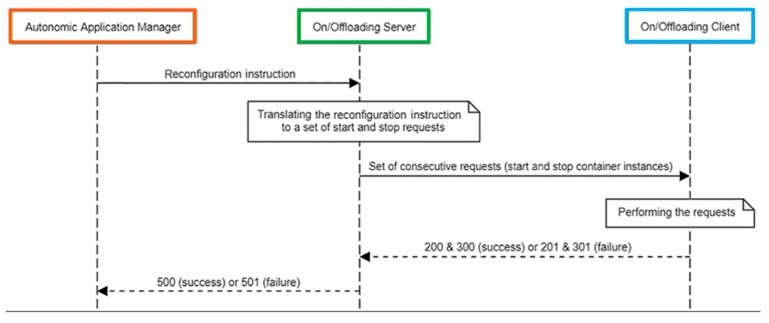
Sequence diagram for an on/offloading scenario performed by the capillary computing architecture.

Figure 4 shows a sequence diagram for a typical scenario in which an instruction is issued by the Autonomic Application Manager for the reconfiguration of the application at runtime. Subsequently, the reconfiguration instruction is translated to a set of consecutive requests by the On/Offloading Server. Each of these requests sent to the On/Offloading Client can be terminating a running container or instantiating a new container. For every request, the On/Offloading Client returns a result value which indicates success or failure of the request execution. If a request is successfully performed by the On/Offloading Client, the return value will be 200 or 300 according to the type of request that can be start or stop a container instance, respectively. If the execution of a request fails due to an error, the return value will be 201 or 301 according to the request’s type, which can be start or stop a container instance, respectively. If the whole set of successive requests which are aimed to perform the reconfiguration instruction issued by the Autonomic Application Manager is successfully accomplished, the return value sent back by the On/Offloading Server will be 500. Otherwise, if the execution of the reconfiguration instruction fails, the return value will be 501.

In order to have a zero failure rate achieved by the sequence diagram shown in Figure 4, start-up times of container instances need to be taken into consideration when Microservices should be offloaded from one resource to another one. In essence, any container termination on the source node before the time when the new container instance on the destination node would be ready to offer its own service means the death of this specific service for a while. Therefore, either success or failure status of start and stop requests should be observed to reach a fortunate on/offloading operation.

## 4. Quality of Service Parameters for Autonomic Orchestration

Application-level monitoring is able to measure parameters which present information about the status of the application such as response time [61]. Violating the service response time threshold may be a consequence of different reasons. A possible cause can be a movement of the IoT device from a geographic location to another place. In this case, the Microservice running on the current Edge node which provides the service is not able to offer favorable QoS required for the smart IoT application. Therefore, a new Microservice needs to be instantiated on another Edge node near the IoT device. A different reason for violations of service response time constraint may also be the situation where the Edge node is overloaded due to an increasing workload.

In all such scenarios when a container-based Microservice should be offloaded from one resource to another one, our Autonomic Application Manager is responsible for choosing the optimal place in order to deploy the Microservice. The new place can be a resource whether on the Edge, Fog or Cloud layer. The deployment decision is made by using a whole range of QoS parameters, which are categorized into two different groups: (i) Network-related parameters and (ii) infrastructure-related parameters, as summarized in Table 2.

Smart IoT application providers can exploit their own pluggable customized node selection method for the container orchestration used by the Autonomic Application Manager. This is because the method employed for choosing the optimal place used to deploy container-based Microservices may depend on the specific use case. Each of the parameters listed in Table 2 may have different levels of significance in various use cases. For instance, Network Jitter (NJ) is the most important parameter for applications such as video conferencing streaming, therefore NJ should have a bigger weight than other parameters in this case as it has more influence on user experience.

In order to provide a desired application QoS, different node selection methods to deploy containers have been proposed so far in different research works. For instance, most of the parameters mentioned are used in a node selection method for the file upload service presented by Stankovski et al. [62]. In their work, the idea is to dynamically deploy each container on the optimal place in a way that the container is instantiated, served and finally destroyed for each request to upload a file.

### 4.1. Network-Related Parameters

The idea of orchestrating containerized Microservices between Edge, Fog and Cloud has raised an important concern about the network quality of the connection across such an advanced computing framework. To this end, different network-related parameters should be considered to improve the application performance. These parameters analyzed for network measurement are categorized into two groups: Static and dynamic. Static parameters are independent from the execution environment at runtime, and they are steady all the time such as the network bandwidth assigned to a host on the Fog or Cloud; in contrast, dynamic parameters vary particularly depending on runtime changes in running conditions such as the quality of network connection between IoT sensors and the server on the Fog or Cloud layer.

#### 4.1.1. Static Network-Related Parameters

Number of Hops (NH): As the number of routers called hops between an IoT device and a host on the Fog or Cloud increases, the negative effect of other traffic on the network quality of the path through which the data flows tends to become notable. Besides that, a predominant part of the network transmission time is the queuing delay at intermediate routers. This is why choosing an effective network configuration with regard to the number of routers between an IoT device and a host on the Fog or Cloud is significant.

Network Bandwidth (NB): It is the maximum amount of data which can be transferred per second through a network interface on the host. The limitation of bandwidth may cause a negative impact on the quality of time-critical and real-time applications as it specifies the ability of the host in terms of the required time to be spent in the transfer of the data to the network instance.

#### 4.1.2. Dynamic Network-Related Parameters

Packet Loss (PL) is the percentage of packets that failed to be transferred through a network path in order to reach the Edge, Fog or Cloud from the IoT device. Network Throughput (NT) implies the rate of successful data delivery across a network connection. Network Delay (ND) determines how long a packet takes to travel across a link from the IoT device to the host on the Edge, Fog or Cloud. Network Jitter (NJ) represents the fluctuation in the end-to-end time delay of sequential packets received by the host. Jitter is significant for real-time services as it affects the size of the active data stream buffers.

### 4.2. Infrastructure-Related Parameters

Each Microservice which needs to be deployed has its own minimum hardware requirements for the resource. These requirements are the minimum number of CPUs, the minimum amount of memory, and the minimum disk space, which are required for deployment. Furthermore, infrastructure-level metrics such as average CPU, memory and disk utilization can significantly affect the service performance at runtime. Therefore, it is essential to exploit a comprehensive monitoring system capable of addressing the whole spectrum of deployment requirements with regard to different parameters. These parameters may be either static as they may be fixed during the execution time, or dynamic since they depend on the status of runtime environment such as changing workloads.

#### 4.2.1. Static Infrastructure-Related Parameters

Hardware characteristics of a resource which can be employed to run a specific Microservice are considered as static infrastructure-related parameters. These parameters are the Number of CPUs (NC), Amount of Memory (AM), and Size of Disk space (SD).

#### 4.2.2. Dynamic Infrastructure-Related Parameters

Monitoring of resources used to run Microservices is critical. Performance optimization may be best obtained by continuously monitoring the usage of CPU (Percentage of CPU usage: PC), memory utilization (Percentage of Memory usage: PM), and disk (Free Disk capacity: FD) over time. For example, a resource with enough amount of available processing capacity, free memory, unused disk space addressing the minimum hardware requirements is significant to be considered at the deployment time of a Microservice.

## 5. Empirical Evaluation

### 5.1. Use Case

In order to demonstrate our proposed capillary distributed computing architecture, we rely on an advanced smart application used for car automation [13]. To this end, a telematics Microservice is deployed on a Motorhome Artificial Intelligence Communication Hardware (MACH) system as the Edge node installed in the car. This telematics system uses various sensor data and stores their measured values on the MACH Edge node. The system also exploits advanced AI methods to analyze the collected data and to trigger run-time alerts in order to notify the logistic center on situations, when possible accidents may occur because of the driver’s frequent maneuvers. The developed Microservice can be used to recognize four types of unexpected driving dynamics including sudden acceleration, hard braking, aggressive right turn and aggressive left turn.

Various situations may arise in which case computations provided on the MACH Edge node has to be offloaded to the Fog. A reason for this may include a sudden logistics computational workload achieving a greater energy efficiency, particularly when the car is running on batteries. Another cause may be providing adequate QoS to the applications, e.g., if the CPU runs out of free cycles, or the memory and data storage on the MACH node are exhausted, and similar. At all such events, Microservices should be elastically offloaded from the Edge node to a specific Fog node in the close proximity of the car. Under such a condition, the Edge node operates as intermediary to receive and pre-process the measured values from sensors, and transmit the data to the Fog node for processing and data storage.

### 5.2. Experimental Design

The purpose of our experimental design was to verify the functionality of our capillary computing architecture, in that it is able to continue providing the telematics service if an event may occur because of which the Microservice should be offloaded from the MACH Edge node to the Fog in the close proximity of vehicle. The MACH system is useful not only for analyzing the telematics data very fast, but also storing measured values at the extreme Edge of the network. Therefore, a specific situation, where the free disk capacity on the MACH Edge node is not available anymore and hence it is not able to provide data storage operation, was considered as the event for which the telematics Microservice movement from the MACH Edge node to the Fog should be approached. This is because on the Fog node, there is enough storage capacity, which is more than the Edge node to store all sensor measurements. In this regard, the threshold for the free storage capacity is set to 100 MB; that means if the free storage capacity on the MACH Edge node is less than 100 MB, the offloading action needs to be performed at runtime.

We run a demonstration that analyzes data collected by sensors during an actual driving trip. A 25-km-long motorway was chosen to collect measured data taken from a regular petrol car on a sunny day. The whole trip took 13 min and 16 s.

Two different types of infrastructures, a set of two proximal Fog nodes and a distant centralized Cloud resource, were used to host the telematics Microservice in order to evaluate the importance of placement decisions made by our proposed capillary computing architecture.

The experiments were evaluated through different important properties achieved during execution: (i) The 99th percentile of the response time; (ii) median response time; (iii) average response time; (iv) standard deviation of response time; and (v) number of detected events. In this use case, the response time represents the system’s reaction time, which means the period of time from sensor data acquisition to when the system recognizes whether a notification has to be sent to the logistic center or not.

All these important properties offered by our proposed capillary computing architecture were compared with another experiment’s results achieved by a basic method which simply redeploys the Microservice from the Edge node to a fixed centralized Cloud instead of Fog.

Each experiment was repeated for five iterations to achieve the average values of important properties and to verify the obtained results and thus to achieve a greater validity of results. Accordingly, the results reported in this work are mean values over five runs for each experiment.

### 5.3. Experimental Setup

**Figure 5:**
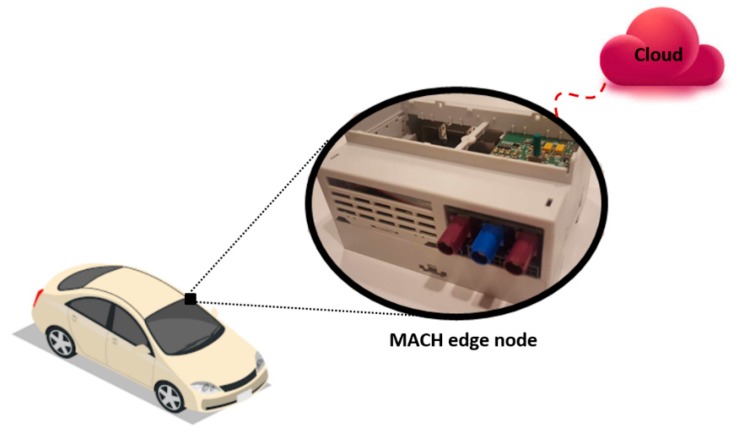
MACH Edge node developed in one of our ongoing projects called OPTIMUM is settled in the vehicle.

In this use case, the core infrastructure used as the Edge resource is installed in the vehicle. The Edge node is an Embedded System (ES) named MACH (Motorhome Ai Communication Hardware), shown in Figure 5, which is developed in one of our ongoing projects called OPTIMUM [63]. Currently, it is successfully installed in prototype motorhome vehicles of the Adria Mobil Company [64]. Therefore, all sensors (e.g., accelerometer and magnetometer, etc.) are connected to MACH at the Edge of the network.

**Figure 6:**
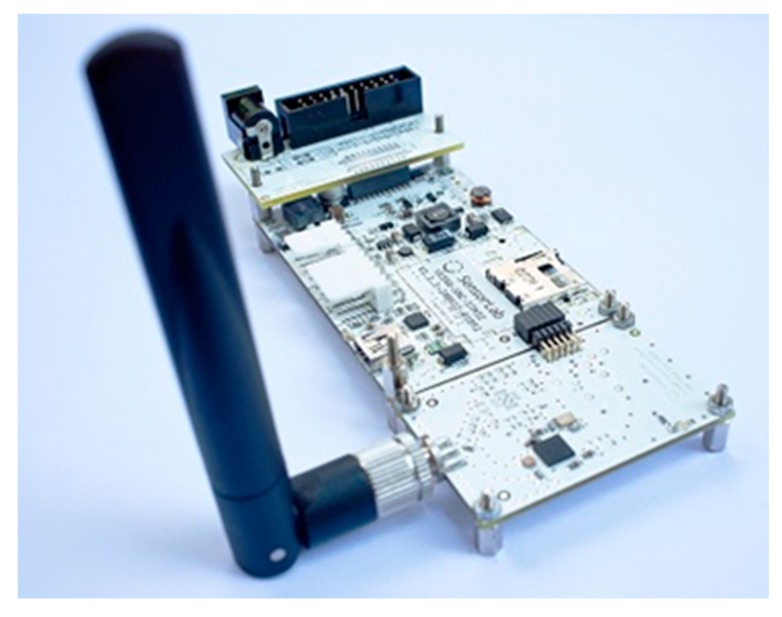
VESNA high-performance microcontroller.

MACH includes a Raspberry Pi 3 model B with a 20 GB storage disk and also a custom extension called VESNA [65] which is able to communicate with different hardware devices through various protocols such as Controller Area Network (CAN) as a robust vehicle bus standard. VESNA, shown in Figure 6, is a fully flexible, modular, high-performance platform for the implementation of Wireless Sensor Networks (WSNs) developed at the Jozef Stefan Institute (JSI), Slovenia.

Free Disk (FD) metric is measured continuously by a Monitoring Agent running on the MACH Edge node to avoid any issue in providing the data storage service. Monitoring Agent periodically sends the measured values of FD metric to the Monitoring Server. As explained before, the free storage capacity threshold which notifies the necessity of offloading the telematics Microservice from the MACH Edge node is set to 100 MB. It should be noted that the possibility of occupying the whole last free 100 MB disk capacity during 10 s in our use case is non-existent. Therefore, while the monitoring interval may be defined as very short in milliseconds, it was set to 10 s to decrease the communication traffic load and any monitoring overhead.

Table 3 shows the characteristics of infrastructures applied in our experiments. The Fog nodes belong to the Academic and Research Network of Slovenia (ARNES), which is a non-profit Cloud infrastructure provider, and the Cloud node belongs to the Faculty of Electrical Engineering, University of Ljubljana (UL). Moreover, all components defined as parts of both the Capillary Container Orchestrator and the Edge/Fog/Cloud Monitoring System were deployed together on a dedicated JSI Server, described in Table 3. These consistently running components, which are explained in the Architecture and design Section, are the Monitoring Server, TSDB, Alarm-Trigger, Scheduling Controller, Autonomic Application Manager, Autonomic Resource Manager and On/Offloading Server.

Sensor measurements such as 3-axis acceleration values are sampled at the rate of 10 Hz where each sample is recorded every 100 ms. Hence, there are approximately 8000 samples for the whole trip. Sampling period was set to 100 ms since this time interval sufficiently offers the system an accurate view of the vehicle’s movements. A sampling interval which is bigger than 100 ms may result in missing vehicle’s dynamics in the running environment, and thus the system may not be agile enough to recognize undesirable movements of the vehicle body, which can lead to dangerous driving situations. On the other hand, a smaller interval will not provide any further information advantage than a 100 ms interval.

### 5.4. Experimental Results

**Figure 7:**
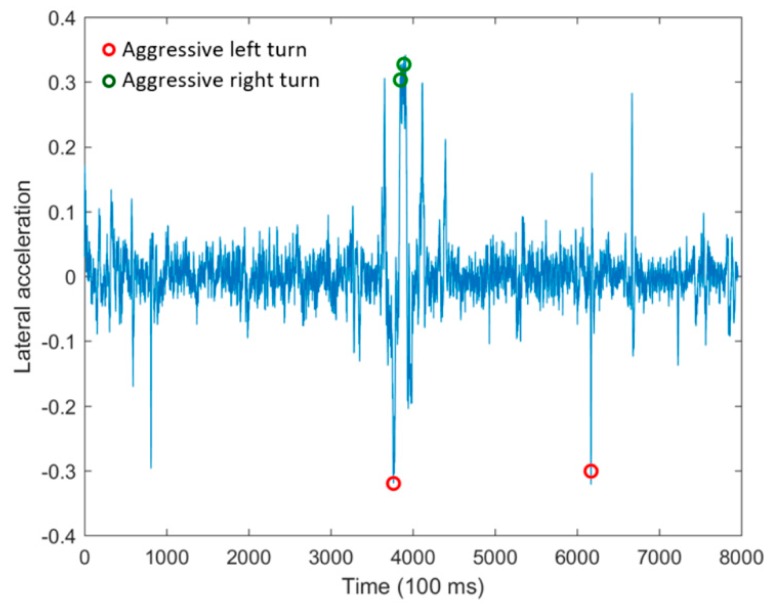
Situations when aggressive left and right turns are recognized.

During the trip, six driving events were recognized by our developed telematics application and their associated notifications were sent to the logistic center at run-time. Figure 7 shows the vehicle’s lateral acceleration taken from sensors during the driving trip. The two circles colored in red demonstrate situations where our developed telematics system recognized aggressive left turns, and two green circles also represent points where aggressive right turns were identified.

**Figure 8:**
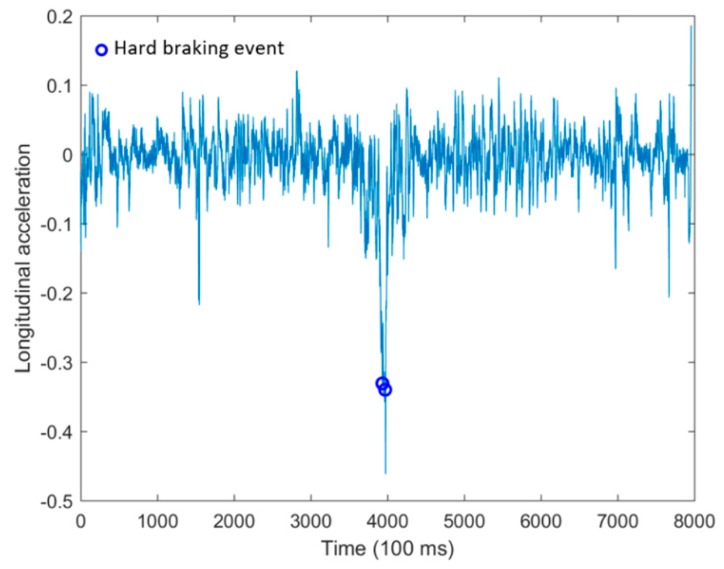
Situations when hard braking events are recognized.

Besides that, the blue circles shown in Figure 8 depict two hard braking events recognized according to the longitudinal acceleration during the driving trip. No sudden acceleration happened during the whole driving trip as well.

In this experiment, we introduced Formula (1) as a node selection method used by the Autonomic Application Manager in our capillary computing architecture. This method chooses the optimal node among all possible Fog nodes based on only three dynamic network-related parameters including PL, NT and ND. For other applications, the deployment decision can be made by exploiting another node selection method in a different way based upon the use case. It should be noted that the list of QoS parameters which can be considered for the autonomic orchestration of Microservices is already presented in Table 2. According to Formula (1), the Fog node with the greater value of Node Selection Rank (NSR) will be chosen to host the service when the offloading action needs to be performed. In this formula, a smaller value of ND as well as PL which is a percentage between 0 and 100 will lead to a better NSR. In contrast, a bigger value of NT will result in a higher NSR.

(1) $$\mathrm{NSR}=\frac{\left(1-\frac{\mathrm{PL}}{100}\right)*\mathrm{NT}}{\mathrm{ND}}$$

**Figure 9:**
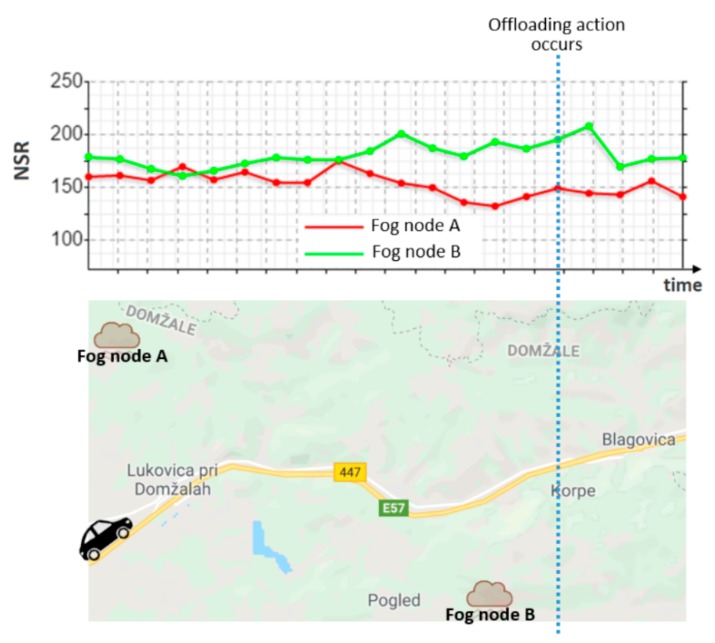
NSR values offered by two Fog nodes in a specific part of the trip.

The NSR value calculated by the proposed formula can imply how Fog nodes are able to provide network performance at runtime. Figure 9 shows NSR values provided by two Fog nodes in a specific part of the trip in our experiment during which an offloading action occurs. During the first half of the road in this figure, the car has almost the same distance from two different Fog nodes called Fog node A and Fog node B. This is the main reason that the NSR provided by both Fog nodes were nearly identical in the first half of the road in this figure. However, during the second half of the road in this figure, the car has almost less distance from Fog node B than Fog node A. This is why Fog node B offers a greater value of NSR in the second half of the road in Figure 9. Therefore, when the offloading action happens to re-deploy the telematics Microservice from the MACH Edge node to the Fog, Fog node B is chosen to host the service since it offers a bigger NSR than Fog node A exactly before the offloading action occurrence time.

**Figure 10:**
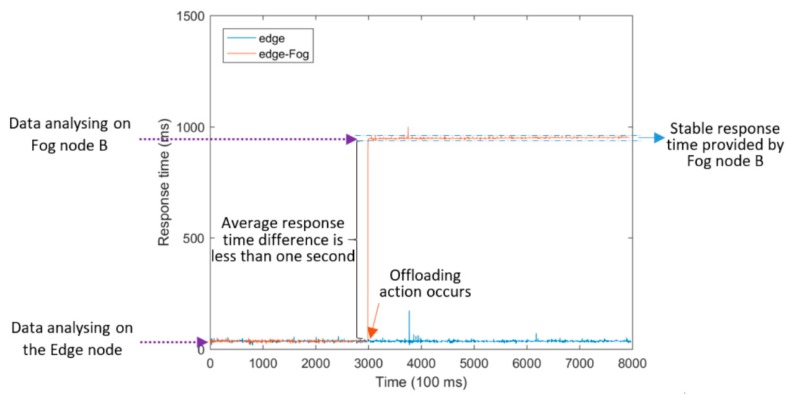
Service response time provided by our proposed capillary computing architecture.

Figure 10 shows the response time provided in two different experiments: (i) Edge and (ii) Edge-Fog. The first one entitled “Edge” shows the execution of the service running only on the Edge node in the whole conducted experiment during which no offloading action happened. The second one entitled “Edge-Fog” shows the experiment in which an offloading action was performed at second = 3000 in order to re-deploy the telematics Microservice from the MACH Edge node to Fog node B. Therefore, in the second experiment called “Edge-Fog”, the service was running on Fog node B after the time when it was offloaded from the MACH Edge node.

**Figure 11:**
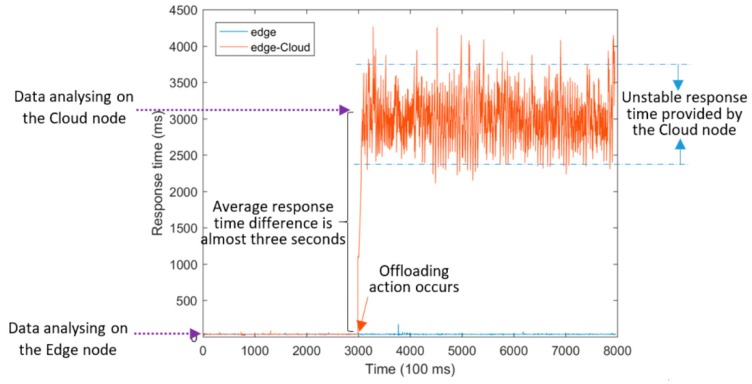
Service response time provided by the unintelligent method.

Similarly, another experiment entitled “Edge-Cloud” was accomplished, as shown in Figure 11. Here, the Cloud node which is introduced in the Subsection “Experimental setup” was used to host the service when an offloading action was executed.

Table 4 presents important properties achieved during execution in all different experiments mentioned above.

Values presented in Table 4 show that Edge computing provides a fast response time for such telematics application and it is able to extract useful information through real-time data analytics at the extreme Edge of the network, in comparison with a Fog or Cloud computing framework. Generally, the average and median response time offered on the Edge was around 35 ms, whereas the average and median response time provided by Fog and Cloud was nearly 1 and 3 s, respectively. The difference between response times provided by Edge and Fog is almost less than one second in experiments. However, this service response time difference is around three seconds for the Cloud.

The 99th percentile value of response time, listed in Table 4, is an applicable indicator for the comparison of delivering QoS in different experiments according to a Service Level Agreement (SLA) widely used in the real-world Cloud computing systems. It is clear that relatively weaker application performance was provided by Cloud compared with Fog in terms of the 99th percentile values. It was almost 1 s achieved by Fog and almost 4 s provided by Cloud, respectively.

Furthermore, it can be simply concluded that the Fog node belonging to ARNES provides a stable quality of infrastructure, since the average response time provided by this node was nearly steady during the entire conducted experiment. This is why the standard deviation of response time achieved on the Fog node is suitably less than 3 ms. On the other hand, drastic fluctuations appear in the response time offered by the application running on the centralized Cloud node which belongs to UL. This fact resulted in the large standard deviation values observed in response time provided by Cloud.

**Figure 12:**
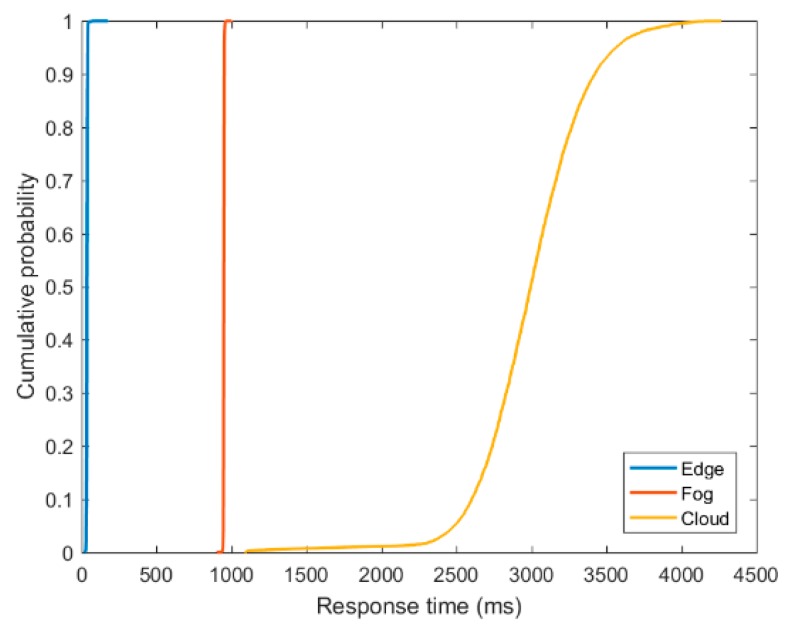
CDF of response time offered by the Edge, Fog and Cloud infrastructures.

The cumulative distribution function (CDF) of the response time provided by the Edge, Fog and Cloud infrastructures is shown in Figure 12. This figure, considered as further confirmation of aforementioned results, shows that both Edge and Fog nodes provided a steady and fast application response time compared to the Cloud-based resource. The probability that the response time offered by the Edge node would be slow is almost zero, whereas the service running on the Fog node performs better than the service running on the Cloud as it has a higher probability to offer desired response time, and thus support the QoS of the application.

In essence, inappropriate infrastructure quality provided by the faraway centralized Cloud node negatively affects the response time at run-time. It should be noted that runtime properties of Cloud-based infrastructures change over time and depend on many factors such as resource reliability and availability. This is why each IaaS provider offers Cloud-based infrastructures with different levels of stability in running conditions over time. Therefore, tracking the reliability of the underlying Cloud infrastructures is essential in order to identify any deterioration of system health.

Moreover, instability of infrastructures may be a consequence of many other reasons related to bandwidth quality such as improper network configuration or network congestion. One particular reason for this is that the application performance may be affected by the network communication quality between the Edge node and the node on the Cloud. In our experimental setup, the path through which data flows between the Edge node and the Fog node includes only three hops, whereas there are 11 hops to the Cloud node. Along this line, not only the number of hops from the Edge node to the destination where the service is running should be taken into account, geographic distance also has an important role in providing a stable and fast application response time.

## 6. Conclusions

Recently, the perspective of smart IoT environments has significantly evolved since these types of applications are becoming more and more time-sensitive, deployed at decentralized locations and their QoS is volatile due to changing conditions such as varying workload or moving IoT devices at runtime [66]. A promising paradigm shift is emerging from the traditional centralized Cloud computing model to distributed Edge and Fog computing frameworks in order to address such challenging scenarios.

Along this line, this paper proposed a distributed computing architecture that includes an Edge/Fog/Cloud Monitoring System and a Capillary Container Orchestrator that is able to handle highly dynamic IoT environments in which the Edge node may be overloaded at runtime due to an increase in the workload. Thus, the container-based Microservices running on the Edge node will be offloaded to the upper layer called the Fog node. This new architecture is also capable of managing situations where IoT devices are moving from one geographic location to another one. The conducted experiments have demonstrated the benefits of our proposed capillary computing architecture to be able to offload requests from an Edge node to a Fog or Cloud resource, when the Edge node is overloaded and it has a limited amount of computation power or storage capacity at runtime.

Running conditions (such as time-varying processing delays, CPU and I/O load factors, etc.) and QoS properties of Edge, Fog or Cloud infrastructures (such as availability, etc.) may vary at runtime, independent from the workload characteristics and geographic location of the IoT devices. Such variations intrinsic to non-deterministic and non-monotonic decision environment are typical limitations of this research work. Therefore, when a Microservice should be launched and deployed, the application provider is required to ensure that the infrastructure is capable of fulfilling all requirements needed to support the service. In this regard, the performance of running infrastructures also needs to be continuously characterized according to the mentioned features.

The use of a secure message access control is one of the most important current challenges of smart IoT applications. To this end, different technologies such as SmartVeh [67] have been proposed so far in order to achieve a high level of data security, satisfy access policies and support data privacy, especially for vehicular computing services. Thus, an attractive field of further research may be the investigation of technologies supporting security aspects on sensitive data, preferably the ones that are published under the Apache 2 license for smoother customization, and also easier integration into our proposed capillary computing architecture.

We have planned to extend the present Edge computing design to include blockchain technologies. This new idea may offer a greater level of security in the function of the overall system, along with opportunities that are useful for turnkey integration with functionalities from outside the IoT world.

## Figures and Tables

**Table 1:** Container-based vs. VM-based virtualization.

Feature	Containers	VMs
Requirement	Container engine e.g., Docker	Hypervisor e.g., Xvisor
Weight	Lightweight	Heavyweight
Size	Small	Large
Boot Time	Fast	Slow

**Table 2:** List of QoS parameters for autonomic orchestration.

QoS Parameter’s Type	Static/Dynamic	Name	Abbreviation	Measurement Unit
Network-related	Static	Number of Hops	NH	#
		Network Bandwidth	NB	MBps
	Dynamic	Packet Loss	PL	%
		Network Throughput	NT	MBps
		Network Delay	ND	Ms
		Network Jitter	NJ	Ms
Infrastructure-related	Static	Number of CPUs	NC	#
		Amount of Memory	AM	MB
		Size of Disk space	SD	GB
	Dynamic	Percentage of CPU	PC	%
		Percentage of Memory	PM	%
		Free Disk	FD	MB

**Table 3:** Characteristics of infrastructures applied in our experiments.

Feature	Fog Nodes	Cloud Node	Dedicated JSI Server
**OS**	Ubuntu 14.04.5 LTS	Ubuntu 16.04.4 LTS	Ubuntu 16.04.4 LTS
**CPU(s)**	1	1	4
**CPU MHz**	2397.222	2399.998	2659.998
**Cache size**	4096 KB	16,384 KB	6144 KB
**Memory**	4096 MB	4096 MB	16,384 MB
**Disk**	10 GB	10 GB	80 GB
**Bandwidth**	1000 MBps	1000 MBps	1000 MBps
**Provider**	ARNES	UL	JSI

**Table 4:** Properties achieved in all different experiments.

Important Properties Achieved During Execution	Edge	Edge-Fog	Edge-Cloud
Average response time offered before on/offloading event	35.60	35.51	35.44
Average response time offered after on/offloading event	none	948.31	3011.69
Median response time obtained before on/offloading event	35.25	35.25	35
Median response time obtained after on/offloading event	none	948.25	3010.5
99th percentile of the response time provided before on/offloading event	42.25	42.75	44.25
99th percentile of the response time provided after on/offloading event	none	954.60	3901.52
Standard deviation of response time achieved before on/offloading event	3.32	2.92	3.22
Standard deviation of response time achieved after on/offloading event	none	2.62	364.50
Number of detected events	6	6	6
